# Understanding Nonprescription and Prescription Drug Misuse in Late Adolescence/Young Adulthood

**DOI:** 10.1155/2013/709207

**Published:** 2013-01-28

**Authors:** Sasha A. Fleary, Robert W. Heffer, E. Lisako J. McKyer

**Affiliations:** ^1^Department of Psychology, Texas A&M University, MS 4235, College Station, TX 77843, USA; ^2^Department of Health and Kinesiology, Texas A&M University, Dulie Bell, College Station, TX 77843, USA

## Abstract

This study explored the extent to which nonprescription and prescription drugs misuse among adolescents/young adults are related to their perception that it is safer than illicit drugs, ease of access, and lower societal stigma. Adolescents/young adults (*N* = 465; *M*_age_ = 18.57, SD = 0.86) completed an online survey about their nonprescription and prescription drug misuse, other substance use, and correlates of use. Perceived risk, societal stigma, and access to nonprescription and prescription drugs were predictive of misuse. Results support program planners working towards targeting perceived risk and societal stigma in reducing misuse and the need to restrict and monitor access to nonprescription and prescription drugs for adolescents/young adults.

## 1. Introduction

Prescription drugs are becoming the drugs of choice for adolescents and young adults with a reported increase in misuse of 212% from 1992 to 2003 [1]. The Substance Abuse and Mental Health Services Administration [2] and Johnston et al. [3] have reported that the misuse or nonmedical use of prescription drugs has a greater prevalence rate than illicit drugs use with the exception of marijuana use, with the highest prevalence rates being reported among adolescents and young adults. The increased incidence and prevalence of nonprescription and prescription drug misuse among young people led to the Office of National Drug Control Policy's [4] prescription drug abuse prevention campaign to target parents of adolescents in 2008.

Friedman [5] and Manchikanti [6] argued that continued prescription drug misuse among adolescents may be attributed to adolescents' perception that this type of drug use is safer than illicit drugs, the ease of access to these drugs, and lower societal stigma about misuse compared to illicit drugs use. These three variables are directly related to peer influence and are consistent with Kandel's [7] adolescent socialization theory, particularly imitation and social reinforcement. Kandel [7] described peer influence, rather than parent influence, as being the most important influence to adolescents' immediate lifestyle, hence peers being more relevant in understanding adolescents' health risk behaviors. Regarding Kandel's [7] theory, imitation involves adolescents modeling their behaviors and attitudes based on others' behaviors and social reinforcement involves adolescents internalizing and displaying behaviors and attitudes approved by others. Both are applicable to adolescents' perception of prescription drug use being safer than illicit drugs and lower stigma about misuse, since these perceptions are based on attitudes displayed by others in conjunction with adolescents' own beliefs. The theory also applies to ease of access to drugs, such that if parents and peers are engaging in prescription drug use, drugs may be more easily accessible to adolescents. Also noteworthy is the developmental level of adolescents/young adults, and according to E. H. Erikson and J. M. Erikson [8] peers are key counterplayers during this stage of life. Adolescents/young adults are forming their identity which may be based on in-group or out-group values. If adolescents'/young adults' group values involve prescription drug use, then there will be norms for use and ease of access to drugs. Understanding the roles of adolescents' perception that this type of drug use is safer than illicit drugs, the ease of access to these drugs, and lower societal stigma about misuse compared to illicit drugs use may provide additional targetable areas for intervention. Hence, the purpose of this study is to explore the extent to which these variables are predictive of adolescents'/young adults' prescription drug misuse. 

In the health risk behaviors and substance abuse literature, abuse has been shown to be related to perceived risk (PR) such that as PR of substance use increases, use decreases [9–12]. PR of nonprescription and prescription drugs have not been extensively explored with most studies addressing PR of nonprescription and prescription drug misuse in relation to illicit drugs. 

Arria et al. [13] conducted a longitudinal study on perceived harmfulness of stimulants and analgesics in college students and found that 25.2% and 27.8% of students attributed a descriptor of “great risk” to the occasional nonmedical use of stimulant and analgesics misuse, respectively. Additionally, prescription stimulants and analgesics were viewed as less risky than cocaine but more risky than marijuana and binge drinking. The Partnership for a Drug Free America [14] (PDFA) reported that 40% of adolescents viewed prescription medication to be safer than illegal drugs. Additionally, adolescents did not believe that prescription pain relievers were addictive and believed that it was okay to use prescription drugs without a prescription once in a while [14]. Prescription drugs may be perceived as being safer than illicit drugs because they can be legitimately prescribed by doctors and are Food and Drug Administration approved [5, 6]. Manchikanti [6] also reported that the increase in social acceptability of medicating ailments may also be responsible for misperceptions of prescription drug misuse safety. Based on these findings, we hypothesize that PR of prescription drugs will be significantly lower than illicit drugs and that the belief that prescription drugs misuse is safer than illicit drug use will be positively related to nonprescription and prescription drug misuse. Additionally, we hypothesize that PR of misuse will be negatively predictive of misuse.

Another variable mentioned by Friedman [5] as being responsible for the increased prevalence in prescription drug misuse is lower societal stigma about misuse. Like PR, the role of parent and peer approval has been extensively studied in the substance abuse literature but few studies have focused on its relations with nonprescription and prescription drug misuse. Ford [15] found that adolescents who had parents and peers with pro-substance abuse attitudes were more likely to report engaging in prescription drug misuse. Additionally, the PDFA [14] highlighted that 21% of adolescents reported that their parents won't care if they caught them engaging in prescription pain reliever misuse. Researchers also found that adolescents whose friends engage in substance use were more likely to engage in prescription drugs misuse [15–18]. According to the PDFA [14], 33% of adolescents reported that there was less shame attached to using prescription pain relievers than illicit drugs. These findings reiterate imitation and social reinforcement as described in Kandel's [7] adolescent socialization theory and the influence of in-groups and out-groups. We hypothesize that perceived societal stigma (PSS) in the form of peer and parent disapproval and perceived peer misuse would be related to nonprescription and prescription drug misuse. We also hypothesize that, similar to PR, participants will perceive peers to be more approving of prescription drug misuse than illicit drug use.

Ease of access to nonprescription and prescription drugs has been suggested as responsible for the increased prevalence of misuse; however, similar to PR and PSS, few researchers have studied the extent to which this is true. Weyandt et al. [19] found that half the sample they examined reported that prescription stimulants were easily accessible on campus with 21.2% and 9.8% reporting being offered stimulants or purchasing stimulants from other students, respectively. However, they did not explore the extent to which easy accessibility was predictive of misuse. Poulin [20] found that students who reported medical use of stimulants reported giving, selling, and being forced to give their medication to others. Giving and selling prescribed medication to others were positively related to increase in nonmedical stimulant use, thus providing evidence for the role of ease of access in stimulant misuse. Manchikanti [6] identified several modes of access to prescription drugs including internet pharmacies, drug theft, sharing among family and friends, doctor shopping, and improper prescribing. The PDFA [14] also provided evidence for the role of easy access to drugs in the increase in misuse prevalence. According to the PDFA [14], adolescents reported having easy access to prescription drugs via parents' medicine cabinets (62%), others' prescriptions (50%), and from the internet (32%). This provides further evidence for the role of imitation and social reinforcement in prescription drug misuse. Therefore, we hypothesize that ease of access to nonprescription and prescription drugs will be predictive of nonprescription and prescription drug misuse.

Based on Kandel's [7] adolescent socialization theory and the proposals by Friedman [5] and Manchikanti [6] about the role of PR, PSS, and ease of access in the increased prevalence of nonprescription and prescription drug misuse, we hypothesize that these variables will be predictive of misuse. Specifically, PR and PSS will be negatively related to misuse and ease of access will be positively related to misuse.

## 2. Method

### 2.1. Participants

The sample was taken from a public college in the Southwestern US. The sample consisted of 465 college students between the ages of 18 and 24 years (*M* = 18.57, SD = 0.86). The majority of the participants (90.5%) were between 18 and 19 years. Participants were predominantly Caucasian (74%), female (60%), and freshmen (73%). Sixteen percent of participants (*N* = 73) reported some prescription drug misuse and 15% (*N* = 71) reported some nonprescription drug misuse. Note that cough and cold syrup and pills misuse (9%, *N* = 42) were not included in the frequency calculations because the question about cough and cold syrup and pills did not distinguish between prescription and nonprescription drugs. Twenty-six percent of participants (*N* = 119) reported some nonprescription and/or prescription drug misuse. 

### 2.2. Measures

 Misuses of nonprescription and prescription drugs were defined as use of medications to get high. PR was measured by perceived personal risk, which is the extent to which the participant felt they would be at risk of getting sick or hurt if they engaged in nonprescription, prescription, and illicit drug use. PR was measured on a 7-point evaluation scale ranging from *No risk at all (1)* to *Very much at risk (7)*. PSS was participants' self-report on their perceptions about how friends and parents felt about them engaging in nonprescription, prescription, and illicit drug use (i.e., parent and peer disapproval). Parent and peer approval was measured on a 5-point Likert scale ranging from *Strongly Approve (1)* to *Strongly Disapprove (5)*. PSS variables also included participants' perceptions of friends' nonprescription and prescription drug misuse. Friends misuse was participants' assessment of the percentage of friends who engaged in misuse in 10% increments (e.g., 0%, 1%–10%, 11%–20%). To measure accessibility of nonprescription and prescription drugs, participants answered questions on whether their friends brought medications to school for recreational use (access in school) and what nonprescription and prescription drugs were kept in the home. Participants were also asked about their perceptions about the safety of nonprescription and prescription drug misuse in relation to illicit drugs use. 

Nonprescription and prescription drugs misuse and illicit drug use were measures of lifetime use on a 5-point frequency scale ranging from *Never (1)* to *More than 40 times (5)*. For nonprescription and prescription drugs, the question stem was “*Have you ever used the following medications recreationally?*” and for illicit drugs the question stem was “*Have you ever engaged in the following activity?*” Prescription drug misuse was measured for the following classifications of medications; prescription pain (e.g., Vicodin, Codeine, OxyContin, Percocet), prescription stimulants (Adderall, Dexedrine, Ritalin), and prescription sedatives and tranquilizers (Mebaral, Quaaludes, Xanax, Valium (benzodiazepines), Nembutal, Fluoxetine). Nonprescription drug misuse was measured for nonprescription pain relievers (e.g., Tylenol, Motrin, Advil, Aleve, Ibuprofen, Aspirin) and nonprescription sleeping pills. Misuse was also measured for cough and cold syrup and pills; however, no distinction was made between prescription and nonprescription. Illicit drugs included marijuana, methamphetamines, crack or cocaine, and inhalants. Participants also completed a demographic questionnaire.

### 2.3. Procedures

 This study was approved by the Institutional Review Board at the university. Data were collected as part of the “Media influences on health risk behaviors and prescription drug use in young adults” project. Data were collected using a survey developed by the authors specifically for this project; however, portions of the survey (e.g., PR and PSS) were modified from Omori and Ingersoll [21]. College students were recruited from the introduction to psychology course to participate in the study. Participants completed an online survey at a computer lab where workstations were separated by desk partitions. The survey was administered via surveymonkey.com and took approximately 35–50 minutes to complete. Participants received two research credits upon completion of the survey. To ensure participant privacy, respondent identification numbers were assigned. 

### 2.4. Statistical Analyses

 Descriptive statistics were computed (see Table 1). To test for differences in PR and perception of societal stigma for nonprescription and prescription drug misuse versus illicit drug use, paired sample *t*-tests were computed. Pearson correlations were computed to explore the relationship between participants PR and PSS for nonprescription and prescription drug misuse and that of illicit drug use. Nonprescription and prescription drug misuse was dichotomized into nonusers (participants who responded *Never* to the questions) and users (participants who reported any misuse). Binary logistic regressions were calculated to differentiate PR, PSS, and access to nonprescription and prescription drugs on self-reported nonusers and misusers. Demographic variables including age, gender, and ethnicity were controlled for in the first step of the regression analyses. 

## 3. Results

 Results of correlations and paired sample *t*-tests are presented in Table 2. PR of nonprescription and prescription drugs misuse was positively correlated with PR of marijuana and crack or cocaine at the *P* ≤ 0.001. Similarly, peer approval for nonprescription and prescription drug misuse was positively correlated with peer approval for marijuana and crack or cocaine use at the *P* ≤ 0.001. Parent approval for prescription pain, prescription sedatives and tranquilizers, prescription stimulants, and nonprescription sleeping pills was positively correlated with marijuana and crack or cocaine use at the *P* < 0.05 to *P* ≤ 0.001 range. Parent approval for nonprescription pain was significantly positively correlated with approval for marijuana use and uncorrelated with approval for crack or cocaine use. Parent approval for cough/cold syrup was uncorrelated with approval for marijuana use and crack or cocaine use. 

PR of all nonprescription and prescription drugs examined were lower than the PR of crack or cocaine confirming the hypothesis that PR of prescription drugs is lower than that of illicit drugs. Participants also perceived risk from marijuana use to be higher than that from nonprescription pain, cough/cold syrup, and nonprescription sleeping pills misuse. Participants perceived risk from the misuse of prescription pain and prescription sedatives and tranquilizers to be higher than that of marijuana use.

Regarding PSS, participants reported parent disapproval of all nonprescription and prescription drugs misuse as significantly lower than parent disapproval of marijuana and crack or cocaine use. Participants reported that their peer disapproval of nonprescription and prescription drugs misuse was significantly lower than that of crack or cocaine. These results confirm that participants perceived peers and parents to be more approving of nonprescription and prescription drug misuse. Participants' PSS of all the nonprescription and prescription drugs measured with the exception of cough/cold syrups and pills and nonprescription pain medication was higher than that of marijuana use. Participants' perceived peers to be less disapproving of nonprescription pain misuse than marijuana use.

 Results of binary logistic regression analyses predicting nonprescription and prescription drug misuse are presented in Table 3. Regarding prescription pain drugs misuse, Caucasians were more likely to be misusers than African-Americans/Black and Hispanics, while PR and peer disapproval were negatively associated with use. Access to prescription medications at home was positively associated with misuse. For prescription sedatives and tranquilizers, African-Americans/Blacks were less likely to be misuses than Caucasians, and parent disapproval and peer use were positively associated with misuse. PR was negatively related to misuse of sedatives and tranquilizers. Age was positively related to misuse of stimulants while peer disapproval and perceived risk were negatively related to misuse. Regarding nonprescription pain medication, peer use was positively related to misuse. No other predictor variables were related to nonprescription pain medication. Access to nonprescription sleeping pills in the home was positively related to misuse. No other predictor variables were related to nonprescription sleep drugs misuse. For cough/cold syrup and pills, peer disapproval and peer use was negatively and positively related to misuse, respectively. Note that the odds ratio and confidence intervals could not be calculated for Asians for any of the prescription drugs, for Hispanics for nonprescription sleep, and for ethnicity and access in home for cold/cough syrup and pills due to extremely large standard errors.

## 4. Discussion

 As discussed in Friedman [5] and Manchikanti [6], we proposed that PR, perception of illicit drugs being safer than street drugs, PSS, and access to nonprescription and prescription drug misuse would be significantly related to misuse. Our results provide some evidence that these variables provide a plausible explanation for increased prescription drug misuse among adolescents/young adults.

Both PR and PSS of nonprescription and prescription drugs misuse and illicit drug use were positively correlated, suggesting that adolescents/young adults recognize that nonprescription and prescription drugs misuse are risky. These findings also provide evidence for the high correlation of illicit drug use and nonprescription and prescription medication misuse [16, 18, 22]. Specifically, PR for one substance may influence PR for the other substance and subsequent decisions to engage in use of both substances. Additionally, adolescents/young adults may belong to in-groups and out-groups that are consistent in how they perceive substance abuse, for example, a group that disapproves of illicit drug use may also disapprove of prescription drug misuse. These results also provide evidence for social reinforcement and imitation of attitudes proposed in the adolescent socialization theory [7].

PR and PSS of nonprescription and prescription drugs being lower than crack or cocaine but not marijuana highlight an important trend that is somewhat consistent with Arria et al. [13] and should be explored further. Specifically, the potential of a substitution effect whereby adolescents/young adults perceive misuse of nonprescription and prescription drugs as a middle ground between too little and too much risk, as well as the formation of a new sub-culture should be explored. These findings also suggest a need to reevaluate and reconfigure programs targeting marijuana prevention with programs emphasizing PR and stigma (possibly via harsher penalties for use). In addition to reevaluating marijuana programs, more should also be done to increase awareness of the risks associated with nonprescription and prescription drug misuse. The Office of National Drug Control Policy's [4] media campaign targeted parents; however, the findings of this study suggest that adolescents/young adults may benefit from additional campaigns targeting them (adolescents/young adults) and their peers that highlight the risks associated with nonprescription and prescription drug misuse. Addressing misuse in this way may be very effective since it will reduce the amount of unhealthy or inappropriate social reinforcement and imitation of undesirable attitudes regarding prescription drug misuse currently displayed by adolescents/young adults. 

The inconsistency in PR and PSS of nonprescription and prescription drugs when compared to marijuana may also explain why pain medication misuse is second only to marijuana use [23]. The only drugs perceived as being less risky than marijuana were nonprescription pain, nonprescription sleep, and cough and cold medications suggesting that adolescents'/young adults' perception of nonprescription and prescription drugs differ probably due to access or level of control imposed by the Food and Drug Administration. Also noteworthy is the nonsignificant relationship between parent disapproval for cough/cold syrup and pills and marijuana and crack or cocaine. Possible explanations for these nonsignificant relationships include parents not viewing these drugs as risky and worthy of discussion with adolescents, parents not recognizing that these drugs are subject to misuse, adolescents mistaking access for approval, or both parents and adolescent not connecting the relationship between misuse of cough/cold medications and illicit drugs. Future research should explore nonprescription drugs, including cough and cold medication, independently. Though nonprescription drugs are less risky than prescription drugs, it is imperative that programs and campaigns incorporate the dangers associated with the improper use of nonprescription drugs, particularly among adolescents and young adults, instead of focusing solely on prescription drugs.

To predict misuse, demographic variables were first controlled for. Given the restricted age range of our sample, it was interesting that there was a significant age difference for prescription stimulant misusers. We propose that since stimulants are known “study drugs”, older participants may be engaging in more stimulant misuse due to demands of classes. Surprisingly, particularly for pain medication, gender was not a significant predictor of misuse. Several researchers have found that women tend to misuse pain medications more often than men [24, 25]. One possible reason for our nonsignificant results is our definition of misuse. In most studies, misuse is broadly defined as non-prescribed use [24], but because our definition was limited to recreational use we may have screened out the young women who misuse these medications for pain relief rather than getting high.

Consistent with Friedman [5], Manchikanti [6], and Ford [15], PSS was the strongest predictor of all nonprescription and prescription drug misuse with the exception of nonprescription sleeping pills. This reiterates the need for prevention programs to target adolescents/young adults; adolescents/young adults influence each other's decision to engage in use therefore increasing the PR and reducing acceptance of nonprescription and prescription drug misuse in some adolescents and young adults may have a ripple effect. Of particular note is the significantly positive relationship between parent disapproval, peer use, and sedatives and tranquilizers misuse. These findings highlight that though parents may be important and instrumental in limiting access and exposure to medications, their disapproval may have the reverse effect on adolescents/young adults. It also further explains the role of peer groups and peer norms in misuse and further confirms Kandel [7] findings about the role of peers in immediate lifestyle choices adolescents/young adults make. 

PR was only a significant predictor of prescription pain, prescription sedatives and tranquilizers, and prescription stimulants, suggesting that other variables may also be contributing to adolescents and young adults' decision to engage in misuse. PR may still be important but may be interacting with other variables in the decision-making process. Future studies should explore other correlates of misuse and how they interact with PR to influence misuse. Future studies should also replicate these findings using a smaller misuser to nonuser ratio. Noteworthy is the failure of the variable, prescription drug not being safer than illicit drug, to predict misuse. This suggests that PR in general may be more important than risk in relation to other substances for predicting misuse. 

Regarding access to medication, medicine kept in the home was only significantly related with misuse of nonprescription sleeping pills. Separate regression analyses were conducted including access to medicine cabinets in the home and this was not significantly correlated to misuse for any classification of drug. We hypothesize therefore that medicine kept in the home being a significant predictor of misuse may be due to a combination of access and acceptability of use for medical reasons as suggested by Manchikanti [6] and consistent with social reinforcement and imitation [7]. Access in school was only predictive of prescription pain, and the authors suspect that these medications were probably the most accessible medications taken to school. Future studies should be more thorough in their definition in school.

## 5. Limitations and Future Directions

 As mentioned before, this study was restricted to recreational users of nonprescription and prescription medication, these misusers do not encompass all non-medical use therefore generalizations about misuse are limited. Additionally, the findings of this study should be viewed as exploratory due to the restrictedness and non-representativeness of the sample. This study should be replicated using a multilocation community sample. Because of the higher misuser to nonuser ratio, statistical power to detect significant correlates of misuse may have been insufficient; therefore, future studies should replicate these analyses with lower misuse to nonuser ratios. A broader definition of PSS may also be warranted in future studies. Other future directions include using the results from this study as a basis for developing a peer-focused prevention intervention and a prescription drug misuse awareness intervention.

## 6. Conclusions

To conclude, adolescents'/young adults' PR and PSS of nonprescription and prescription drugs misuse differ from their perception of illicit drug use, particularly for crack or cocaine. Their perceptions are important because they are correlated with misuse and program planners should work towards targeting these perceptions to prevent and decrease misuse. Additionally, access to medications also influences misuse and this suggests that to prevent and decrease use, stronger measures should be taken to restrict access to OTC and prescription drugs.

## Figures and Tables

**Table 1:** Descriptive statistics for the sample.

	Users *N* (%)	Age of first use in years *M* (SD)	Perceived risk *M* (SD)	Perceived societal stigma, parent *M* (SD)	Perceived societal stigma, peer *M* (SD)
Prescription drugs					
Prescription pain	50 (10.7)	17.32 (1.48)	5.30 (1.71)	4.38 (.90)	3.93 (1.09)
Prescription sedatives and tranquilizers	22 (4.7)	17.23 (1.66)	5.84 (1.43)	4.71 (.60)	4.23 (.90)
Prescription stimulants	41 (8.8)	18.05 (2.30)	5.06 (1.83)	4.51 (.85)	3.98 (1.09)
Nonprescription drugs					
Nonprescription pain	61 (13.1)	15.18 (2.84)	3.60 (2.00)	2.43 (1.21)	3.19 (1.33)
Nonprescription sleeping pills	22 (4.7)	17.09 (1.60)	4.82 (1.74)	3.95 (1.12)	3.77 (1.11)
No distinction					
Cough/cold syrup	42 (9)	15.33 (3.18)	3.99 (2.00)	2.70 (1.31)	3.42 (1.31)
Illicit drugs					
Marijuana	134 (29.9)	17.78 (1.67)	5.07 (1.90)	4.87 (.48)	3.49 (1.37)
Crack or cocaine	10 (2.1)	17.80 (1.55)	6.76 (.71)	4.97 (.30)	4.73 (.58)

**Table 2:** Significant correlations and mean differences of perceived risk and perceived societal stigma (peer disapproval) of non-prescription and prescription drug misuse with illicit drug use.

	Perceived risk^a^				Perceived societal stigma, peer^a^				Perceived societal stigma, parent^a^			
	Marijuana		Crack or cocaine		Marijuana		Crack or cocaine		Marijuana		Crack or cocaine	
	*r*	MD	*r*	MD	*r*	MD	*r*	MD	*r*	MD	*r*	MD
Prescription drugs												
Pain	.32	.22*	.30	−1.46	.45	.44	.35	−.80	.14**	−.43	.20	−.59
Sedatives and tranquilizers	.37	.77	.38	−.92	.44	.74	.43	−.50	.30	−.10**	.41	−.25
Stimulants	.46	−.01 (ns)	.27	−1.69	.50	.48	.34	−.76	.34	−.30	.31	−.45
Nonprescription Drugs												
Pain	.25	−1.47	.21	−3.15	.34	−.30	.17	−1.54	.12*	−2.38	.07 (ns)	−2.53
Sleeping pills	.31	−.25*	.24	−1.93	.41	.27	.32	−.97	.18	−.86	.18	−1.01
No distinction												
Cough/cold syrup and pills	.49	−1.09	.23	−2.77	.36	−.08 (ns)	.23	−1.31	.08 (ns)	−2.11	.05 (ns)	−2.26

MD: mean difference; ns: nonsignificant.

^a^ *P* ≤ 0.001 unless were indicated, *P < 0.05, **P < 0.01.

**Table 3:** Results of binary logistic regression analyses predicting misuse from risk perception, societal stigma, and access.

	Prescription			Nonprescription		No Distinction
	Pain OR (95% CI)	Sedatives and Tranquilizers OR (95% CI)	Stimulants OR (95% CI)	Pain OR (95% CI)	Sleep OR (95% CI)	Cough/Cold OR (95% CI)
Step 1 {Δ*R* ^2^}	(.06)	(.10)	(.08)	(.02)	(.03)	(.04)

Age	1.06 (.75, 1.49)	1.20 (.76, 1.88)	1.73** (1.27, 2.36)	1.03 (.75, 1.41)	.85 (.47, 1.54)	1.21(.89, 1.66)
Gender	.68 (.37, 1.25)	.59 (.24, 1.44)	.94 (.48, 1.84)	.61 (.35, 1.06)	1.65 (.63, 4.34)	.54 (.28, 1.04)
Ethnicity^a^						
Black	.20** (.07, .56)	.16** (.04, .63)	.50 (.13, 1.88)	2.54 (.33, 19.71)	.75 (.09, 6.02)	.00^b^
Asian	.00^b^	.00^b^	.00^b^	.95 (.05, 16.50)	.74 (.04, 12.95)	.00^b^
Hispanic	.19 (.03, 1.10)	.22 (.02, 2.37)	.18 (.02, 2.114)	3.63 (.37, 36.83)	.00^b^	.00^b^
Other	.30 (.09, 1.03)	.67 (.15, 2.96)	.32 (.06, 1.66)	2.67 (.31, 23.19)	1.02 (.11, 9.92)	.00^b^

Step 2 {Δ*R* ^2^}	(.28)	(.31)	(.31)	(.30)	(.29)	(.27)

Age	1.00 (.66, 1.51)	.90 (.45, 1.80)	2.18*** (1.48, 3.21)	.94 (.65, 1.35)	.77 (.42, 1.40)	1.28 (.89, 1.83)
Gender	.96 (.47, 1.99)	.49 (.15, 1.54)	1.23 (.55, 2.72)	.67 (.35, 1.26)	1.64 (.56, 4.80)	.52 (.25, 1.10)
Ethnicity^a^						
Black	.23* (.07, .76)	.09** (.02, .54)	.66 (.13, 3.41)	2.52 (.29, 21.93)	.38 (.04, 4.11)	.00^b^
Asian	.00^b^	.00^b^	.00^b^	.65 (.03, 13.66)	1.53 (.06, 40.06)	.00^b^
Hispanic	.09* (.01, .72)	.18 (.01, 3.15)	.21 (.01, 3.41)	3.35 (.28, 40.55)	.00^b^	.00^b^
Other	.29 (.07, 1.22)	.48 (.08, 3.07)	.85 (.12, 6.00)	.62 (.25, 1.51)	1.21 (.09, 17.02)	.00^b^
PR	.78* (.64, .96)	.63** (.46, .87)	.71** (.56, .91)	.84 (.67, 1.04)	.91 (.69, 1.20)	1.00 (.79, 1.27)
PSSD	1.42 (.42, 4.89)	.28 (.07, 1.14)	.62 (.19, 2.02)	.62 (.25, 1.51)	.56 (.14, 2.28)	.65 (.23, 1.81)
PSS:						
Parent	.92 (.63, 1.34)	21.03* (1.9, 229.7)	.77 (.52, 1.15)	1.08 (.77, 1.51)	.81 (.49, 1.34)	.93 (.64, 1.35)
Peer	.52*** (.36, .75)	.84 (.47, 1.50)	.56** (.36, .87)	.70 (.48, 1.02)	.69 (.39, 1.19)	.52** (.34, .79)
Peer use	1.12 (.97, 1.32)	1.50** (1.16, 1.94)	1.15 (.98, 1.35)	1.24*** (1.12, 1.36)	1.14 (.89, 1.46)	1.19** (1.06, 1.33)
Access						
School	2.09* (1.01, 4.35)	1.87 (.60, 5.76)	1.54 (.65, 3.63)	1.26 (.64, 2.47)	1.03 (.36, 2.90)	1.55 (.73, 3.30)
Home	1.69 (.84, 3.42)	3.57 (.75, 16.90)	1.14 (.36, 3.57)	.10 (.01, 1.27)	16.5 (3.4, 79.1)***	.00^b^

PR: perceived risk; PSS: perceived societal stigma; PSSD: prescription safer than street drug; Δ*R* ^2^: change in Nagelkerke *R* ^2^ at each step; ^a^White/Caucasian is the comparison group;^ b^odds ratio cannot be computed due to extremely large standard error.

*P < 0.05, **P < 0.01, ***P ≤ 0.001.
